# Supplementary material for: Phylogenomics of the benzoxazinoid biosynthetic pathway of Poaceae: gene duplications and origin of the Bx cluster
Source: BMC Evol Biol. 2012 May 11;12:64. doi: 10.1186/1471-2148-12-64 (PMC3449204; doi:10.1186/1471-2148-12-64)
Supplement: Additional file 1 — Phylogenetic tree and intron map of the CYP71C sequences of Poaceae. [file 1471-2148-12-64-S1.pdf]

Additional file 1

Figure S1 Phylogenetic tree and intron map of the CYP71C sequences of Poaceae rooted with five CYP76 sequences of *Arabidopsis thaliana*. This tree is the expanded view of the tree shown on Figure 2. Presence or absence of the first common intron (at position 192) or of the second intron (at position 336) is marked as "1" or "2" respectively. In the absence of intron, a "0" is indicated. Branch lengths are drawn in scale in terms of the number of substitutions per site. Intron localizations are not marked on root sequences. Only nodes with bootstrap values superior to 75% are shown (100 iterations).

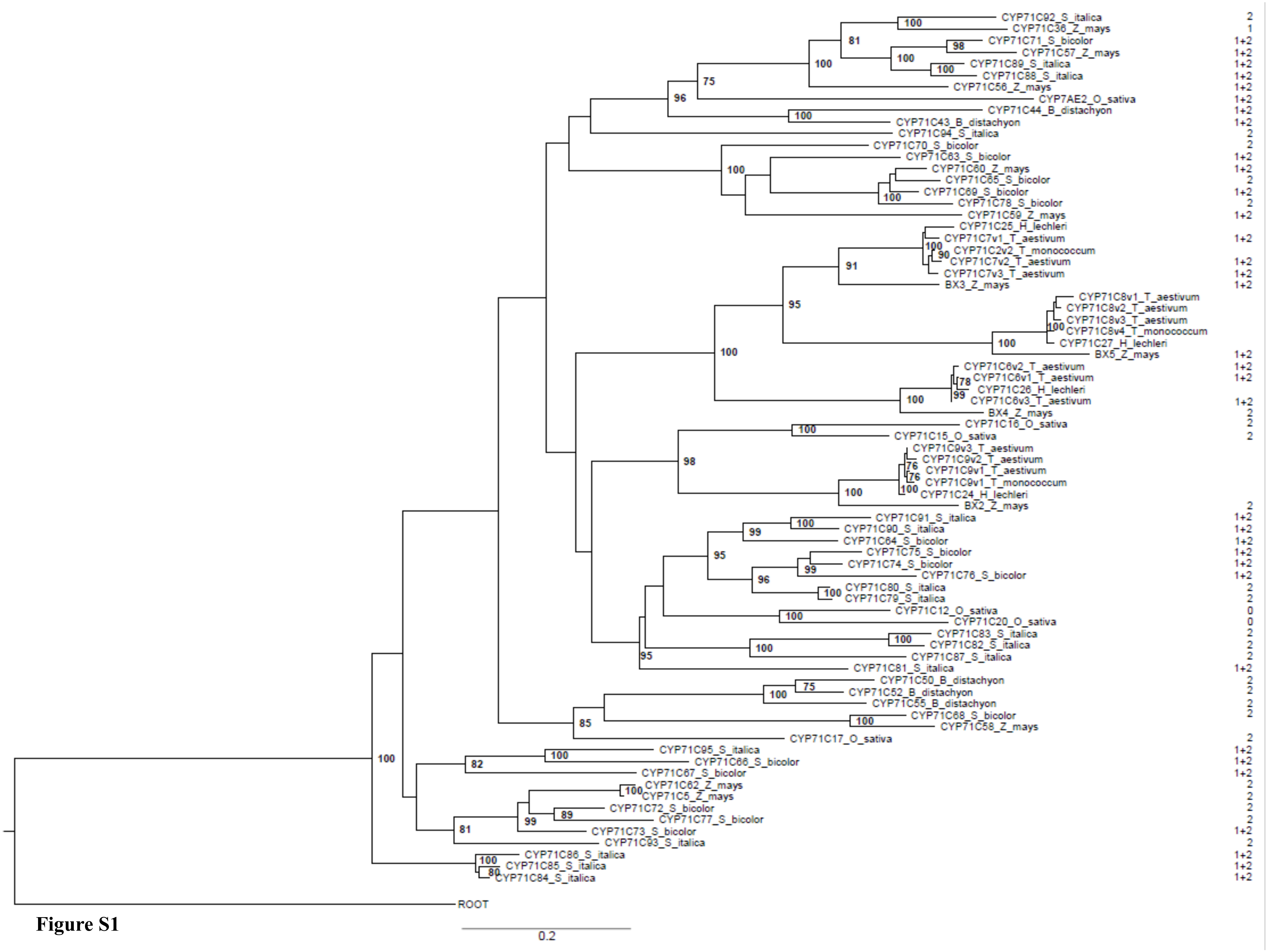
CYP71C92_S_italica
CYP71C36_Z_mays
CYP71C71_S_bicolor
CYP71C57_Z_mays
CYP71C89_S_italica
CYP71C88_S_italica
CYP71C56_Z_mays
CYP7AE2_O_sativa
CYP71C44_B_distachyon
CYP71C43_B_distachyon
CYP71C94_S_italica
CYP71C70_S_bicolor
CYP71C63_S_bicolor
CYP71C60_Z_mays
CYP71C65_S_bicolor
CYP71C69_S_bicolor
CYP71C78_S_bicolor
CYP71C59_Z_mays
CYP71C25_H_lechleri
CYP71C7v1_T_aestivum
CYP71C2v2_T_monococcum
CYP71C7v2_T_aestivum
CYP71C7v3_T_aestivum
BX3_Z_mays
CYP71C8v1_T_aestivum
CYP71C8v2_T_aestivum
CYP71C8v3_T_aestivum
CYP71C8v4_T_monococcum
CYP71C27_H_lechleri
BX5_Z_mays
CYP71C6v2_T_aestivum
CYP71C6v1_T_aestivum
CYP71C26_H_lechleri
CYP71C6v3_T_aestivum
BX4_Z_mays
CYP71C16_O_sativa
CYP71C15_O_sativa
CYP71C9v3_T_aestivum
CYP71C9v2_T_aestivum
CYP71C9v1_T_aestivum
CYP71C9v1_T_monococcum
CYP71C24_H_lechleri
BX2_Z_mays
CYP71C91_S_italica
CYP71C90_S_italica
CYP71C64_S_bicolor
CYP71C75_S_bicolor
CYP71C74_S_bicolor
CYP71C76_S_bicolor
CYP71C80_S_italica
CYP71C79_S_italica
CYP71C12_O_sativa
CYP71C20_O_sativa
CYP71C83_S_italica
CYP71C82_S_italica
CYP71C87_S_italica
CYP71C81_S_italica
CYP71C50_B_distachyon
CYP71C52_B_distachyon
CYP71C55_B_distachyon
CYP71C68_S_bicolor
CYP71C58_Z_mays
CYP71C17_O_sativa
CYP71C95_S_italica
CYP71C66_S_bicolor
CYP71C67_S_bicolor
CYP71C62_Z_mays
CYP71C5_Z_mays
CYP71C72_S_bicolor
CYP71C77_S_bicolor
CYP71C73_S_bicolor
CYP71C93_S_italica
CYP71C86_S_italica
CYP71C85_S_italica
CYP71C84_S_italica
ROOT
0.2

Figure S1
