# Supplementary material for: Phylogenomics of the benzoxazinoid biosynthetic pathway of Poaceae: gene duplications and origin of the Bx cluster
Source: BMC Evol Biol. 2012 May 11;12:64. doi: 10.1186/1471-2148-12-64 (PMC3449204; doi:10.1186/1471-2148-12-64)
Supplement: Additional file 2 — Maize BX2-BX5 alignment showing putative helices, P450 motifs, SRSs and sites under positive selection. [file 1471-2148-12-64-S2.pdf]

Additional File 2

Figure S2. Maize BX2-BX5 alignment showing putative helices, P450 motifs, SRSs and sites under positive selection according to M8 results. In blue are sites potentially under positive selection according to M8 results from codeml analyses. Putative helices are indicated in dotted boxes, SRSs are in orange boxes and the five P450 motifs are in purple.

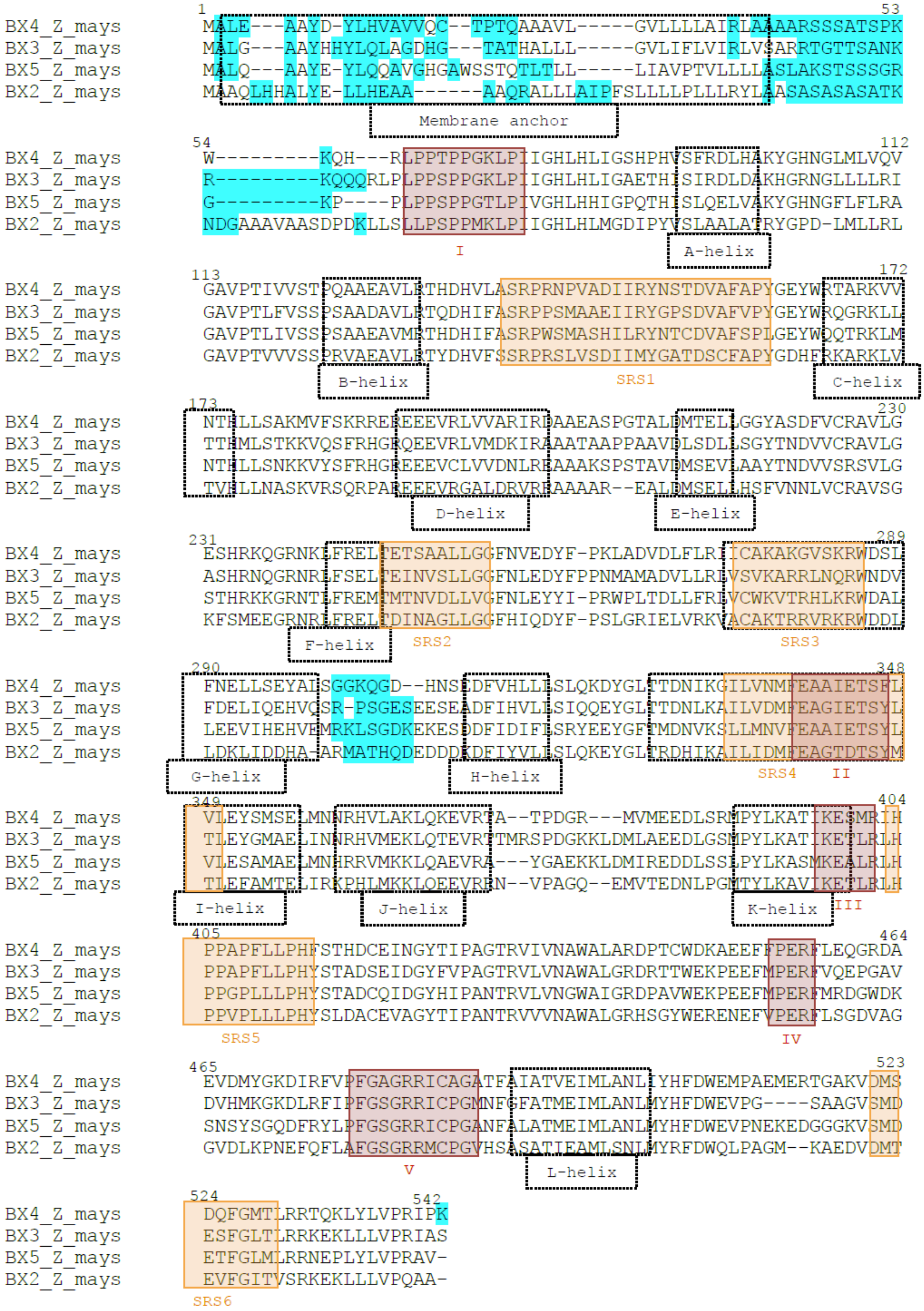
1 53
BX4_Z_mays MALE---AAYD-YLHVAVVQC--TPTQAAAVL-----GVLLLLAIRLAAAARSSSATSPK
BX3_Z_mays MALG---AAYHHYLQLAGDHG---TATHALLL-----GVLIFLVIRLVSARRTGTTSANK
BX5_Z_mays MALQ---AAYE-YLQQAVGHGAWSSTQTLTLL-----LIAVPTVLLLLASLAKSTSSSGR
BX2_Z_mays MAAQLHHALYE-LLHEAA------AAQRALLLAIPFSLLLPLLLRYLAASASASASATK
Membrane anchor
54 112
BX4_Z_mays W---------KQH---RLPPTPPGKLPIIGHLHLIGSHPHVSFRDLHAKYGHNGLMLVQV
BX3_Z_mays R---------KQQQRLPLPPSPPGKLPIIGHLHLIGAETHISIRDLDAKHGRNGLLLLRI
BX5_Z_mays G---------KP----PLPPSPPGTLPIVGHLHHIGPQTHISLQELVAKYGHNGFLFLRA
BX2_Z_mays NDGAAAVAASDPDKLLSLLPSPPMKLPIIGHLHLMGDIPYVSLAALATRYGPD-LMLLRL
I
A-helix
113 172
BX4_Z_mays GAVPTIVVSTPQAAEAVLRTHDHVLASRPRNPVADIIRYNSTDVAFAPYGEYWRTARKVV
BX3_Z_mays GAVPTLFVSSPSAADAVLRTQDHIFASRPPSMAAEIIRYGPSDVAFVPYGEYWRQGRKLL
BX5_Z_mays GAVPTLIVSSPSAAEAVMRTHDHIFASRPWSMASHILRYNTCDVAFSPLGEYWQQTRKLM
BX2_Z_mays GAVPTVVVSSPRVAEAVLRTYDHVFSSRPRSLVSDIIMYGATDSCFAPYGDHFRKARKLV
B-helix
SRS1
C-helix
173 230
BX4_Z_mays NTHLLSAKMVFSKRRERREEEVRLVVARIRDAAEASPGTALDMTELLGGYASDFVCRAVLG
BX3_Z_mays TTHMLSTKKVQSFRHGRQEEVRLVMDKIRAAATAAPPAAVDLSDLLSGYTNDVVCRAVLG
BX5_Z_mays NTHLLSNKKVYSFRHGREEEVCLVVDNLREAAAKSPSTAVDMSEVLAAYTNDVVSRSVLG
BX2_Z_mays TVHLLNASKVRSQRPAREEEVRGALDRVRRAAAAR--EALDMSELLHSFVNNLVCRAVSG
D-helix
E-helix
231 289
BX4_Z_mays ESHRKQGRNKLFRELTETSAALLGGFNVEDYF-PKLADVDLFLRIICAKAKGVSKRWDSL
BX3_Z_mays ASHRNQGRNRLFSELTEINVSLLGGFNLEDYFPPNMAMADVLLRLVSVKARRLNQRWNDV
BX5_Z_mays STHRKKGRNTLFREMTMTNVDLLVGFNLEYYI-PRWPLTDLLFRLVCWKVTRHLKRWDAL
BX2_Z_mays KFSMEEGRNRLFRELTDINAGLLGGFHIQDYF-PSLGRIELVRKVACAKTRRVRKRWDDL
F-helix
SRS2
SRS3
290 348
BX4_Z_mays FNELLSEYALSGGKQGD--HNSEDFVHLLLSLQKDYGLTTDNIKGILVNMFEAAIETSFL
BX3_Z_mays FDELIQEHVQSR-PSGESEESEADFIHVLLSIQQEYGLTTDNLKAILVDMFEAGIETSYL
BX5_Z_mays LEEVIHEHVEMRKLSGDKEKESDDFIDIFLSRYEEYGFTMDNVKSLLMNVFEAAIETSYL
BX2_Z_mays LDKLIDDHA-ARMATHQDEDDDKDFIYVLLSLQKEYGLTRDHIKAILIDMFEAGTDTSYM
G-helix
H-helix
SRS4
II
349 404
BX4_Z_mays VLEYSMSELMNNRHVLAKLQKEVRTA--TPDGR---MVMEEDLSRMPYLKATIKESMRIH
BX3_Z_mays TLEYGMAELINNRHVMEKLQTEVRTTMRSPDGKKLDMLAEEDLGSMPYLKATIKETLRLH
BX5_Z_mays VLESAMAELMNHRRVMKKLQAEVRA---YGAEKKLDMIREDDLSSLPYLKASMKEALRLH
BX2_Z_mays TLEFAMTELIRKPHLMKKLQEEVRRN--VPAGQ--EMVTEDNLPGMTYLKAVIKETLRLH
I-helix
J-helix
K-helix
III
405 464
BX4_Z_mays PPAPFLLPHFSTHDCEINGYTIPAGTRVIVNAWALARDPTCWDKAEEFFPERFLEQGRDA
BX3_Z_mays PPAPFLLPHYSTADSEIDGYFVPAGTRVLVNAWALGRDRTTWEKPEEFMPERFVQEPGAV
BX5_Z_mays PPGPLLLPHYSTADCQIDGYHIPANTRVLVNGWAIGRDPAVWEKPEEFMPERFMRDGWDK
BX2_Z_mays PPVPLLLPHYSLDACEVAGYTIPANTRVVVNAWALGRHSGYWERENEFVPERFLSGDVAG
SRS5
IV
465 523
BX4_Z_mays EVDMYGKDIRFVPFGAGRRICAGATFAIATVEIMLANLLYHFDWEMPAEMERTGAKVDMS
BX3_Z_mays DVHMKGKDLRFIPFGSGRRICPGMNFGFATMEIMLANLMYHFDWEVPG----SAAGVSMD
BX5_Z_mays SNSYSGQDFRYLPFGSGRRICPGANFALATMEIMLANLMYHFDWEVPNEKEDGGGKVSMD
BX2_Z_mays GVDLKPNEFQFLAFGSGRRMCPGVHSASATIEAMLSNLMYRFDWQLPAGM--KAEDVDMT
V
L-helix
524 542
BX4_Z_mays DQFGMTLRRTQKLYLVPRIPK
BX3_Z_mays ESFGLTLRRKEKLLLVPRIAS
BX5_Z_mays ETFGLMLRRNEPLYLVPRAV-
BX2_Z_mays EVFGITVSRKEKLLLVPQAA-
SRS6

Figure S2
